# lincRNA00907 promotes NASH progression by targeting miRNA-942-5p/TAOK1

**DOI:** 10.18632/aging.205730

**Published:** 2024-04-10

**Authors:** Gang Du, Zhaochen Jiang, Tong Xia, Mingkun Liu, Zeyang Liu, Huaxin Zhou, Hao Zhang, Xiangyu Zhai, Bin Jin

**Affiliations:** 1 Organ Transplant Department, Qilu Hospital of Shangdong University, Jinan 250012, China; 2 Department of Hepatobiliary Surgery, The Second Hospital of Shangdong University, Jinan 250033, China

**Keywords:** NASH, lincRNA00907, TAOK1, miRNA-942-5p

## Abstract

Objective: The study aims to examine the involvement of lincRNA00907 in the advancement of non-alcoholic steatohepatitis (NASH).

Methods: The examination was conducted to assess the expression of linc00907 in liver tissues from NASH patients and healthy individuals. High-fat diets induced NASH in mouse models, while palmitic acid/oleic acid treatment was used to create *in vitro* cell models. Various techniques, such as qRT-PCR, Oil Red O staining and gene knockdown/overexpression, were used to assess the impact of linc00907 on genes related to lipid metabolism and immunity, as well as intracellular lipid accumulation. Furthermore, dual-luciferase reporter assays were carried out to confirm the connection between miRNA-942-5p and linc00907 or TAOK1 mRNA.

Results: Linc00907 was found to be significantly upregulated in both NASH patients and NASH mouse models. Overexpression of linc00907 led to an increase in intracellular lipid accumulation, while knockdown of linc00907 resulted in decreased lipid content. It was found that miRNA-942-5p binds with linc00907, and their interaction was confirmed in dual-luciferase reporter assays. Additionally, TAOK1 was predicted to be a downstream target of miRNA-942-5p, and the upregulation of TAOK1 due to linc00907 was reversed by miRNA-942-5p overexpression. linc00907 overexpression reduces apoptosis but can be reversed by TAOK1 knockdown. The reduction of TAOK1 counteracted the impact of linc00907 on gene expression associated with lipid metabolism and immunity, as well as on the accumulation of intracellular lipids.

Conclusions: Our research suggests that linc00907 functions as a competitive endogenous RNA (ceRNA) by sequestering miRNA-942-5p, thus increasing the expression of TAOK1 and encouraging lipid accumulation in hepatocytes, leading to the aggravation of NASH development. Targeting the linc00907/miRNA-942-5p/TAOK1 axis may hold therapeutic potential for the treatment of NASH.

## INTRODUCTION

Non-alcoholic fatty liver disease (NAFLD) is a prevalent hepatic disorder characterized by fat accumulation in the liver in the absence of excessive alcohol consumption [1]. NAFLD may advance to a more severe form of the disease called non-alcoholic steatohepatitis (NASH), which is connected with inflammation, injury to liver cells, and fibrosis [2, 3]. Ultimately, NASH can cause cirrhosis, liver failure, and liver cancer, which are major public health problems [4, 5].

NASH is a multifaceted process, caused by both genetic and environmental factors [6]. Recent findings propose that long intergenic non-coding ribonucleic acids (lincRNAs) have significant implications in regulating gene expression and the pathogenesis of NASH and other different disorders involving the liver [7–9]. lincRNAs can facilitate the synthesis of metabolic products such as cholesterol and fatty acids within hepatocytes, thereby increasing intracellular lipid accumulation and contributing to the development and progression of NASH [10, 11].

lincRNA00907, also referred to as long intergenic non-protein coding RNA 907, is a newly discovered lincRNA which has not been extensively characterized functionally [12]. Earlier research has indicated its participation in different illnesses, such as cancer, cardiovascular disorders, and neurological disorders [13, 14]. Nevertheless, its contribution to liver diseases, primarily NASH, is still mostly unexplored.

MicroRNAs (miRNAs) are small, non-coding RNAs that regulate the expression of genes post-transcriptionally by binding to the 3’ untranslated regions (3’UTRs) of target mRNAs, causing either degradation or translational repression. The dysregulation of miRNAs has been linked to the development and progression of various diseases, such as liver disease. Previous research has identified miR-690 and miR-122 as an essential agent in NASH as it is depleted in NASH patients and guards against hepatic steatosis and inflammation [15, 16].

Recently, there has been heightened emphasis on the functional interconnections between lincRNAs and miRNAs. It has been suggested that lincRNAs function as competing endogenous RNAs (ceRNAs) by absorbing miRNAs, ultimately regulating the expression of miRNA objective genes [17]. CeRNA crosstalk between lincRNAs and miRNAs has been implicated in various diseases, such as cancer and cardiovascular disorders [18, 19]. However, the role of lincRNA00907 in this ceRNAs regulatory network within the context of NASH has not been extensively explored. In addition, lincRNAs may also facilitate the progression of NASH through mechanisms involving the activation of signaling pathways or the regulation of RNA-binding proteins [20, 21].

In this investigation, our aim was to examine the function of lincRNA00907 in NASH advancement and its possible interplay with miRNA-945. This, in turn, leads to the progression of NASH. Our hypothesis was that lincRNA00907 might act as a ceRNA, sequestering miRNA-945 and resulting in the amplification of its target gene TAO kinase 1 (TAOK1). To validate our hypothesis, we initially assessed the expression levels of lincRNA00907 in liver tissues from both healthy controls and NASH patients. Our findings indicate a considerable increase in lincRNA00907 expression in NASH patients, implying its probable involvement in disease progression.

To investigate the functional importance of lincRNA00907 in NASH, we performed *in vitro* experiments with human hepatic cell lines. We observed that lincRNA00907 overexpression resulted in TAOK1 upregulation whereas its knockdown caused downregulation. Furthermore, a potential ceRNA regulatory mechanism was proposed as we discovered a reciprocal correlation between the levels of expression of lincRNA00907 and miRNA-945.

## MATERIALS AND METHODS

### Cell culture

THLE-2 cells, obtained from the American Type Culture Collection (ATCC, Manassas, VA, USA), were cultured under standard conditions (37° C, 5% CO^2^). THLE-2 cells were cultured in Dulbecco’s Modification of Eagle’s Medium (DMEM, Gibco, Carlsbad, CA, USA) containing 10% foetal bovine serum (FBS, Gibco). For passaging, cells were trypsinized, centrifuged, and the pellet was resuspended in fresh medium. Prior to experimentation, cells were tested for mycoplasma contamination using a Mycoplasma Detection Kit (Merck, Burlington, MA, USA), following the manufacturer’s instructions. The details of cell-specific assays and experiments will be described in the subsequent sections of the manuscript.

### Clinical sample collection

NASH tissue samples were collected from patients diagnosed with NASH through pathological examination following liver biopsy. Additionally, normal liver tissues were obtained from healthy individuals who underwent unrelated surgeries and did not have any liver disease. Informed written consent was obtained from all participants following the Declaration of Helsinki, and the study protocol was approved by the Institutional Review Board of Second Hospital of Shandong University. Upon collection, liver tissues were immediately snap-frozen in liquid nitrogen and transported to the laboratory for subsequent processing.

### RNA sequencing

Total RNA was extracted from the snap-frozen liver tissues using the RNeasy Mini Kit (Qiagen, Venlo, The Netherlands) per manufacturer’s instructions. Quantity and quality of the extracted RNAs were assessed using the NanoDrop 2000 (Thermo Fisher Scientific, Waltham, MA, USA) and Agilent 2100 Bioanalyzer (Agilent Technologies, Santa Clara, CA, USA), respectively. RNA libraries were prepared using the TruSeq RNA Library Prep Kit v2 (Illumina, San Diego, CA, USA) and sent for high-throughput sequencing on the Illumina NextSeq 500 platform. The sequencing data generated were then processed and analysed as detailed in subsequent sections.

### RNA extraction

Total RNA was extracted using the TRIzol reagent (Invitrogen, Waltham, MA, USA), following the manufacturer’s standard protocol. In brief, tissues or cells were homogenized in TRIzol, followed by phase separation using chloroform, and RNA precipitation with isopropanol. The RNA pellet obtained was washed with 75% ethanol and dissolved in RNase-free water. The concentration and purity of the extracted RNA were quantified using the NanoDrop 2000 spectrophotometer (Thermo Fisher Scientific).

### qRT-PCR

Linc00907 expression in tissues or cells was detected using qRT-PCR assays. RNA was reversed to synthesize cDNA using a reverse transcription kit (Vazyme, Nanjing, China). Bio-Rad (Bio-Rad, Hercules, CA, USA) equipment and SYBR Mix (Vazyme) were used for qRT-PCR. The primer sequences for linc00907 were as follows: forward primer: 5’- CATCCAGACAGCCAAGCTCA-3’ and reverse primer: 5’-CAGTGCTGGGTCCAATCTGT-3’.

### Establishment of NASH mouse model

A NASH mouse model was developed by feeding 8-week-old male C57BL/6 mice (Jackson Laboratory, Bar Harbor, ME, USA) a lipotoxic high-fat and high-cholesterol (HFHC) diet or high-fat diet (HFD) for 16 weeks. The control group was maintained on a standard chow diet. Both groups had free access to water. NASH progression was monitored by assessing body weight, liver weight, biochemical analysis, and histological examination every four weeks. At the end of the 16 weeks, mice were anaesthetized with isoflurane, and liver tissues were obtained to assess the extent of NASH through histological analysis, as well as to extract RNA for high-throughput sequencing. All animal experiments were conducted in accordance with the guidelines approved by the Animal Care and Use Committee of the Shandong University Institute.

### Cellular NASH model

To induce the cellular NASH model, cells were exposed to a mixture of palmitic acid (PA) and oleic acid (OA). The cells were treated with a specific concentration of PA (250 μM) and OA (500 μM). The PA was initially dissolved in ethanol while OA in 0.01M sodium hydroxide (NaOH) as a stock solution. This stock solution was then diluted with 10% BSA to achieve the required final concentrations for treatment. Post-treatment, intracellular lipid accumulation was assessed by staining the cells with Oil Red O.

### Oil Red O staining

Cell and tissue sections were initially fixed in a 10% formaldehyde solution. This was followed by a thorough rinse with phosphate-buffered saline (PBS). Subsequently, the fixed samples were incubated in a 0.5% Oil Red O solution. This lipid-soluble dye is known to selectively stain neutral triglycerides and lipids within the cells and tissues. After a sufficient incubation period, the sections were rinsed with distilled water to remove any unbound dye. The slides were then mounted for subsequent microscopic examination.

### Plasmid transfection

Plasmid transfection was conducted using Lipofectamine 2000 with cells at 70-80% confluency. Per microgram of plasmid DNA, 2.5 μl of Lipofectamine 2000 was used. After incubation, DNA-Lipofectamine 2000 complexes were added to the cells. Post-transfection, cells were incubated for 48 hours for optimal gene expression.

### Adenovirus-mediated knockdown

The following double-stranded shRNA sequences were used: linc00907 shRNA sequence, forward, 5’- CACCGGGAGATCTAGTTTCCCATCCCGAAGGATGGGAAACTAGATCTCCC -3’; reverse, 3’- CCCTCTAGATCAAAGGGTAGGGCTTCCTACCCTTTGATCTAGAGGGAAAA -5’. Cells were prepared in a suitable growth medium at 70% confluency. Following this, infection was carried out with recombinant adenoviruses that express short hairpin RNA (shRNA) specifically targeting the linc00907. After a 48-hour incubation period at 37° C in a 5% CO2 atmosphere, cells were harvested and assessed for successful knockdown using downstream applications such as quantitative PCR.

### Adenoviral-mediated liver linc00907 knockdown

Adenoviruses carrying the specific shRNA sequence targeting the gene of interest were used for *in vivo* knockdown. Adult mice were intravenously injected, via the tail vein, with the adenoviral constructs. Post-injection, mice were monitored for any adverse reactions and allowed to recover under controlled conditions. Efficiency of the gene knockdown in the liver was assessed using qPCR.

### Dual-luciferase reporter assay

To validate the interactions between the target RNA and miRNA, dual-luciferase reporter assays were performed. The 3’UTR of the target RNA containing the suspected miRNA binding sites was cloned downstream of the firefly luciferase gene in the psiCHECK-2 vector (Promega, Madison, WI, USA). Co-transfection of the reporter construct and miRNA mimics into HEK293 cells was carried out using Lipofectamine 2000 transfection reagent. After 48 hours of incubation at 37° C, luciferase activity was assessed using the Dual-Luciferase Reporter Assay System (Promega). Firefly luciferase activity served as the experimental readout, while Renilla luciferase activity acted as the internal control to normalize transfection efficiency. Reduced firefly luciferase activity in the presence of miRNA mimics indicated a direct interaction between the miRNA and cloned 3’UTR [22].

### Statistical analysis

Statistical analyses (unpaired Student *t* test) were conducted using GraphPad Prism version 5 (GraphPad, Boston, MA, USA). Statistically significant differences were assumed with *P< 0.05, **P<0.01, ***P<0.001.

## RESULTS

### Differential expression and functional analysis of lincRNAs in NASH

**Figure 1:**
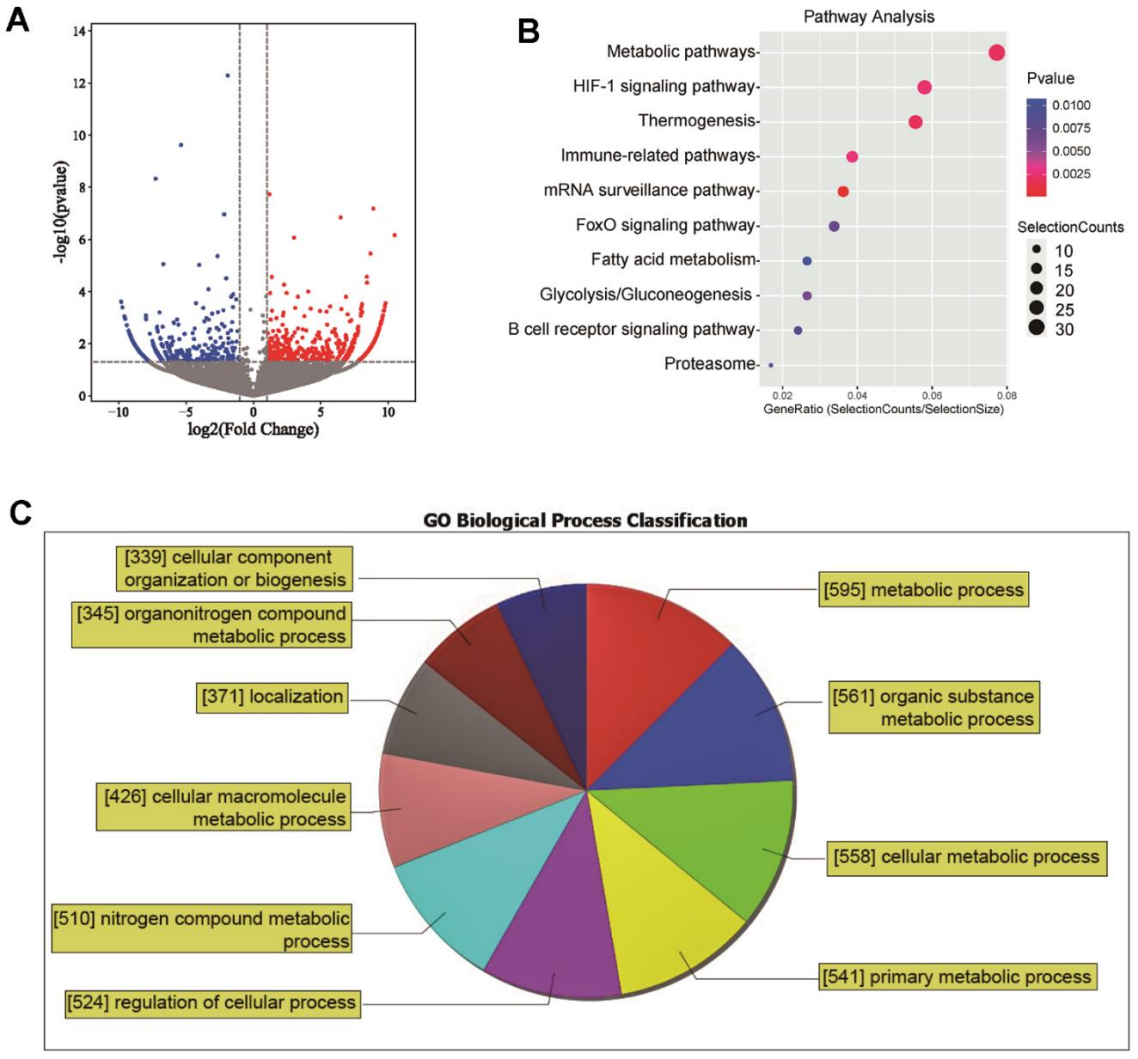
**Differential expression and functional analysis of lincRNAs in NASH.** (**A**) A volcano plot of differentially expressed lincRNAs; (**B**) KEGG enrichment analysis of differentially expressed lincRNAs; (**C**) GO Functional analysis of the differentially expressed lincRNAs.

Through high-throughput transcriptome sequencing of three NASH patients and three normal liver samples, a significant number of differentially expressed lincRNAs were identified. Of these, 997 lincRNAs were upregulated and 808 were downregulated in NASH tissues. These findings were visually represented in a volcano plot (Figure 1A). Further analysis was pursued to understand the broader biological implications of these differential expressions. The differentially expressed genes were subjected to a Kyoto Encyclopedia of Genes and Genomes (KEGG) enrichment analysis. When sorted by P-value and the number of enriched genes, it was observed that the differentially expressed lincRNAs were primarily enriched in metabolic pathways, HIF-1 signaling pathway, thermogenesis, and various immune-related pathways (Figure 1B). Functional analysis of the differentially expressed lincRNAs using Gene Ontology (GO) revealed that 595 lincRNAs were involved in metabolic processes. Approximately 47% of the lincRNAs were found to be involved in biological functions related to metabolic process (Figure 1C). These results highlight the profound involvement of lincRNAs in the pathogenesis of NASH.

### Upregulation of linc00907 correlates with NAFLD and NASH

**Figure 2:**
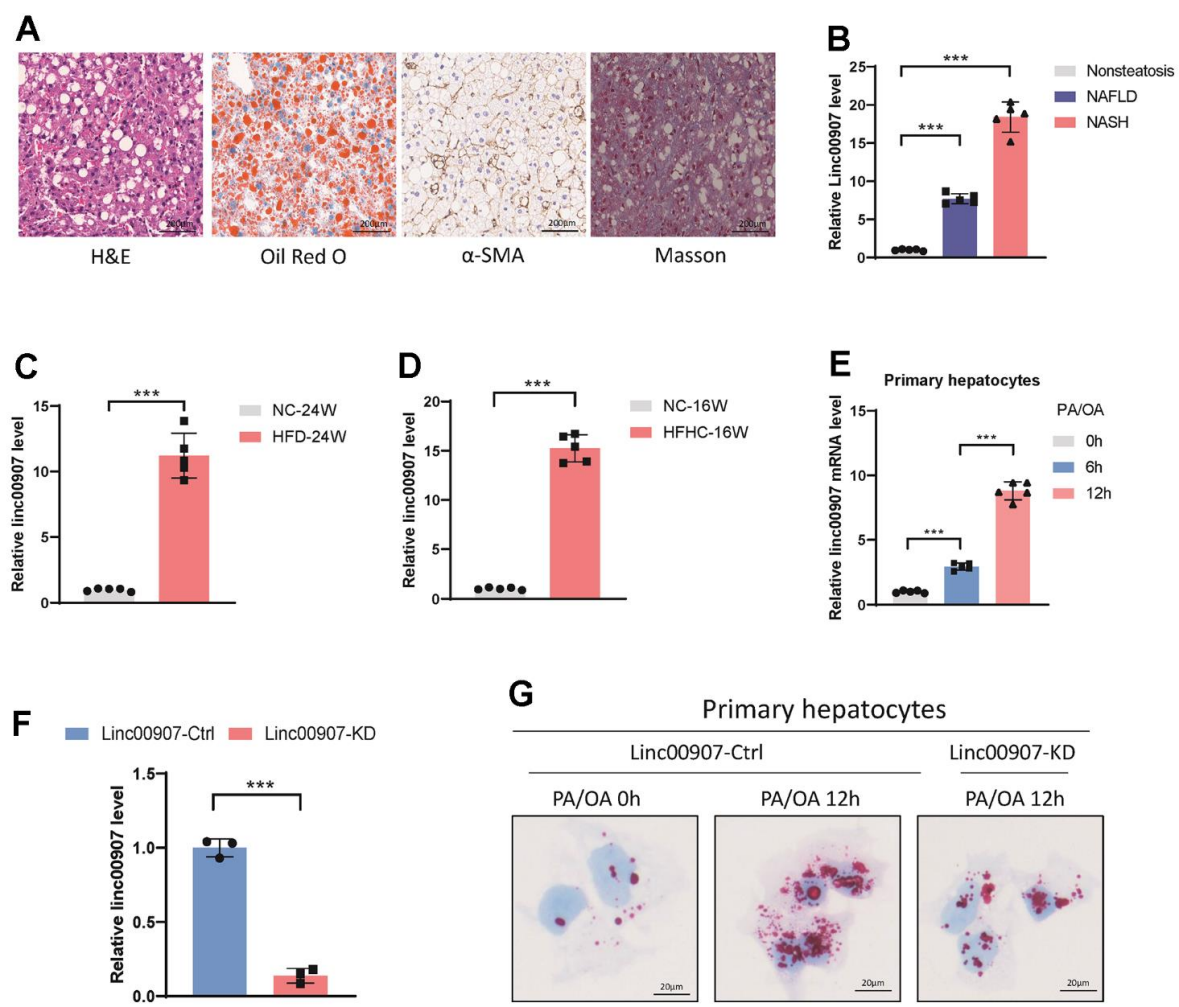
**linc00907 upregulates in NAFLD and NASH.** (**A**) Representative images of H&E, Oil Red O, α-SMA and Masson staining of liver sections from NASH mouse model; (**B**) linc00907 upregulates in both NAFLD and NASH; (**C**, **D**) linc00907 upregulates in NASH conditions established via HFD (**C**) and HFHC (**D**) as opposed to normal mouse liver tissues; (**E**) linc00907 upregulates in the NASH cellular model; (**F**) qRT-PCR analysis has confirmed the effective knockdown of linc00907 within the cells; (**G**) lipid accumulation was significantly mitigated upon knockdown of linc00907.

We successfully generated mouse models of NAFLD and NASH (Figure 2A). A comparative analysis of the expression levels of linc00907 in these models and normal mouse livers revealed a distinct upregulation of linc00907 in both NAFLD and NASH conditions, with the latter demonstrating a more pronounced expression (Figure 2B). Further investigations involving NASH mouse models established via HFD and HFHC consistently indicated an augmented expression level of linc00907 in NASH conditions as opposed to normal mouse liver tissues (Figure 2C, 2D). To corroborate these findings in a cellular context, a NASH cellular model was developed using PA/OA. Consistent with observations from the animal models, we discovered an elevated expression of linc00907 in the NASH cellular model (Figure 2E). Intracellular lipid content, assessed through Oil Red O staining, significantly increased after stimulation with PA/OA and showed a correlation with the duration of PA/OA treatment. Interestingly, this lipid accumulation was significantly mitigated upon knockdown of linc00907 (Figure 2F, 2G). Collectively, these results establish a direct positive association between the severity of NASH, both *in vitro* and *in vivo*, and the overexpression of linc00907. Importantly, the reduction in intracellular lipid content following linc00907 knockdown highlights its potential as a therapeutic target for NASH.

### linc00907 modulates cellular lipid accumulation

**Figure 3:**
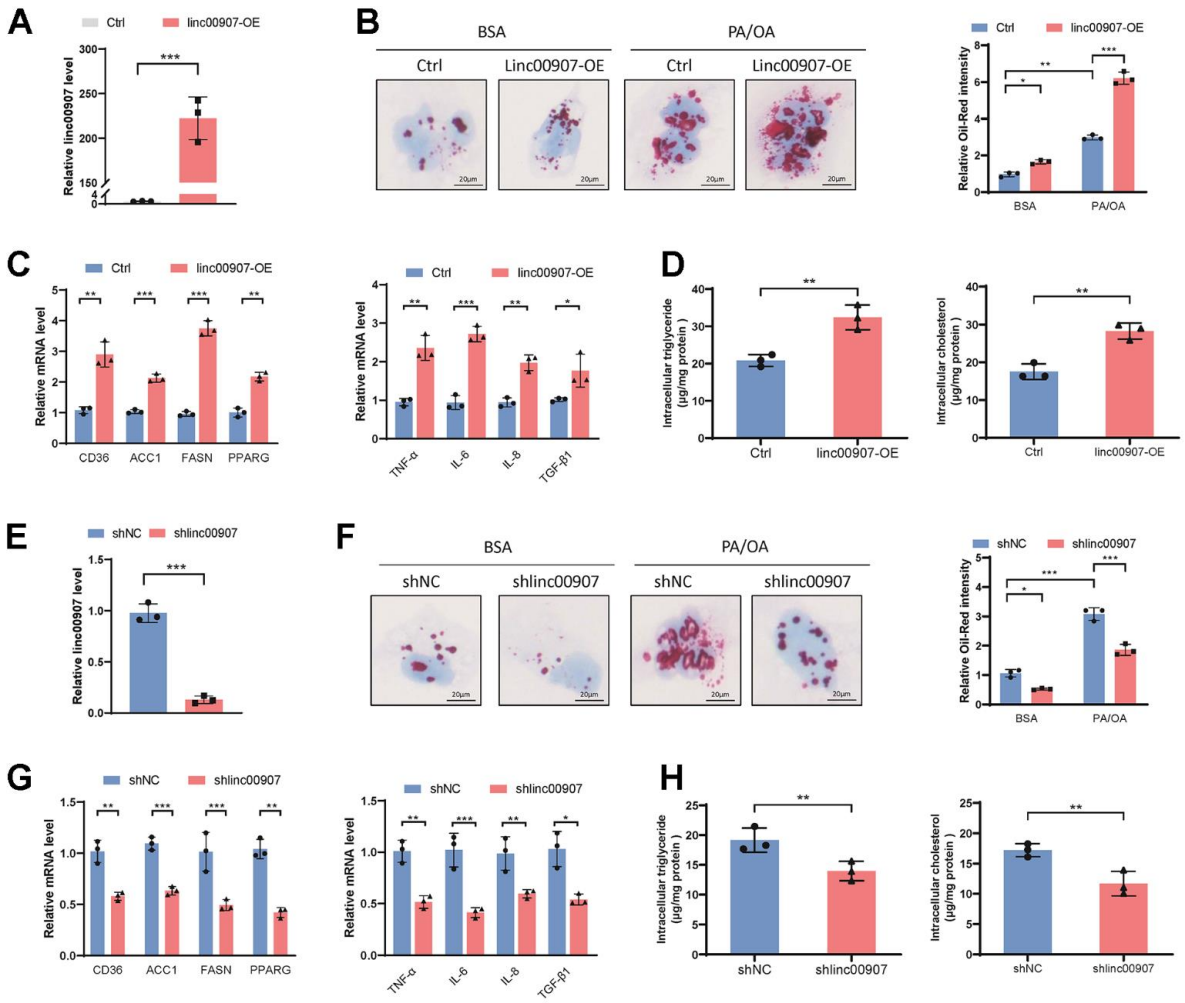
**linc00907 modulates cellular lipid accumulation.** (**A**) Linc00907 plasmids were transfected to construct a THLE-2 cell line with overexpressed linc00907; (**B**) overexpression of linc00907 increased intracellular lipid content; (**C**) overexpression of linc00907 significantly elevated the expression of FASN, CD36, ACC1, TNF-α and TGF-β1; (**D**) linc00907 overexpression resulted in elevated intracellular levels of triglycerides and cholesterol; (**E**) construction of linc00907 knockdown THLE-2 cell line using adenovirus shRNA; (**F**) knockdown of linc00907 markedly reduced lipid content in the NASH model; (**G**) knockdown of linc00907 downregulated the expression levels of lipid synthesis-related genes and immune-related genes; (**H**) knockdown of linc00907 resulted in decreased intracellular triglycerides and cholesterol levels.

Our results elucidate the role of linc00907 in cellular functions through a series of cellular experiments. Initially, we transfected linc00907 plasmids constructing an overexpressed linc00907 THLE-2 cell line (Figure 3A). Oil Red O staining revealed that overexpression of linc00907 increased intracellular lipid content. Moreover, when a NASH model was induced in these overexpressed cells using PA/OA, they demonstrated an even greater lipid accumulation (Figure 3B). Gene expression levels of lipid metabolism-associated genes and immune-related genes were measured through qRT-PCR. We found that overexpression of linc00907 significantly elevated the expression of fatty acid synthase (FASN), CD36, acetyl-CoA carboxylase 1 (ACC1), tumor necrosis factor alpha-like (TNF-α) and transforming growth factor beta 1 (TGF-β1) genes (Figure 3C). Additionally, linc00907 overexpression resulted in elevated intracellular levels of triglycerides and cholesterol (Figure 3D). To further comprehend the role of linc00907, we constructed linc00907 knockdown THLE-2 cell line using adenovirus shRNA (Figure 3E). Knockdown of linc00907 markedly reduced lipid content in the NASH model, and downregulated the expression levels of lipid synthesis-related genes and immune-related genes (Figure 3F, 3G). Similarly, the knockdown of linc00907 resulted in decreased intracellular triglycerides and cholesterol levels (Figure 3H). Taken together, these results suggest that linc00907 can upregulate the expression of lipid synthesis and immune-related genes, thereby increasing intracellular lipid content.

### Knockdown of linc00907 alleviates NASH symptoms in HFHC diet mouse model

**Figure 4:**
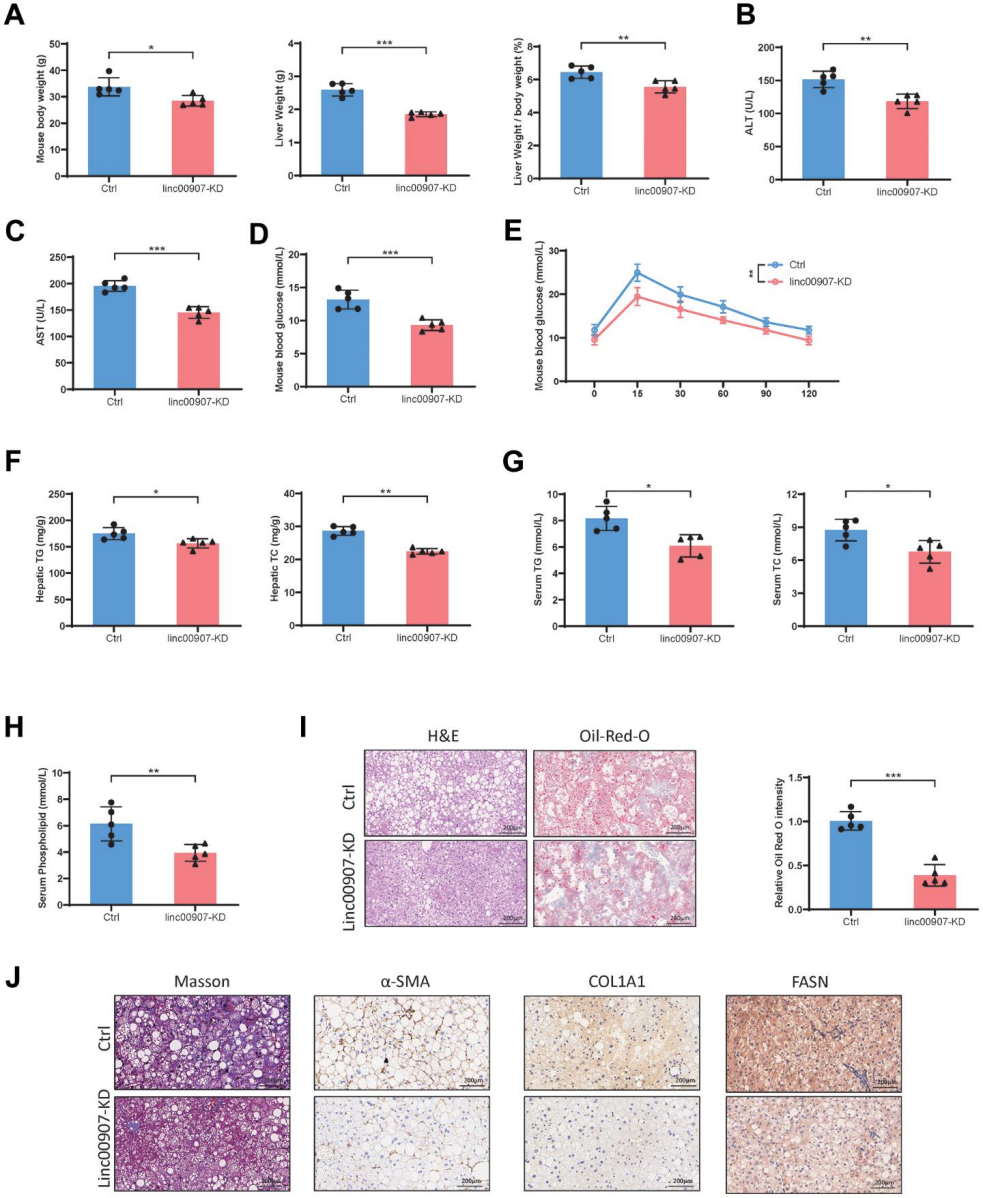
**Knockdown of linc00907 alleviates NASH symptoms in HFHC diet mouse model.** (**A**) Linc00907 knockdown results in lower body and liver weights, with a reduced liver/body weight ratio; (**B**) linc00907 knockdown leads to a reduction in serum ALT levels in mice; (**C**) linc00907 knockdown leads to a reduction in serum AST levels in mice; (**D**) mice in the knockdown group displayed lower blood glucose levels; (**E**) the OGTT confirmed higher glucose tolerance in linc00907 knockdown mice; (**F**) total triglyceride and total cholesterol levels in liver were lower in the knockdown group; (**G**) TG and TC levels in blood were lower in the knockdown group; (**H**) the serum phospholipid levels were also lower in the knockdown group; (**I**) H&E and Oil Red O staining of liver tissues; (**J**) α-SMA, Masson, COL1A1 and FASN staining of liver sections.

We further investigated the impact of linc00907 on NASH in an animal study. Initially, we suppressed the expression level of linc00907 in mouse liver by tail vein injection of AAV-shlinc00907. Subsequently, both knockdown and control groups of mice were subjected to establish HFHC diet NASH models. Compared to the control group, mice with linc00907 knockdown exhibited lower body and liver weights, with a reduced liver/body weight ratio (Figure 4A). The levels of hepatic injury markers, aspartate aminotransferase (AST) and alanine aminotransferase (ALT), were also lower in the knockdown group (Figure 4B, 4C). Mice in the knockdown group displayed lower blood glucose levels than the control group, and the oral glucose tolerance test (OGTT) further confirmed higher glucose tolerance in these mice (Figure 4D, 4E). Upon examination of total triglyceride and total cholesterol levels in both liver and blood, we found that both were lower in the knockdown group compared to the control group (Figure 4F, 4G). Additionally, the serum phospholipid levels were also lower in the knockdown group (Figure 4H). Histological examination using H&E, α-SMA, Masson, COL1A1 and FASN staining revealed that the degree of hepatic steatosis and hepatocellular ballooning in linc00907 knockdown mice was less severe than that in the control group, and lipid accumulation was also less pronounced (Figure 4I, 4J).

### linc00907 interacts with miR-942-5p

**Figure 5:**
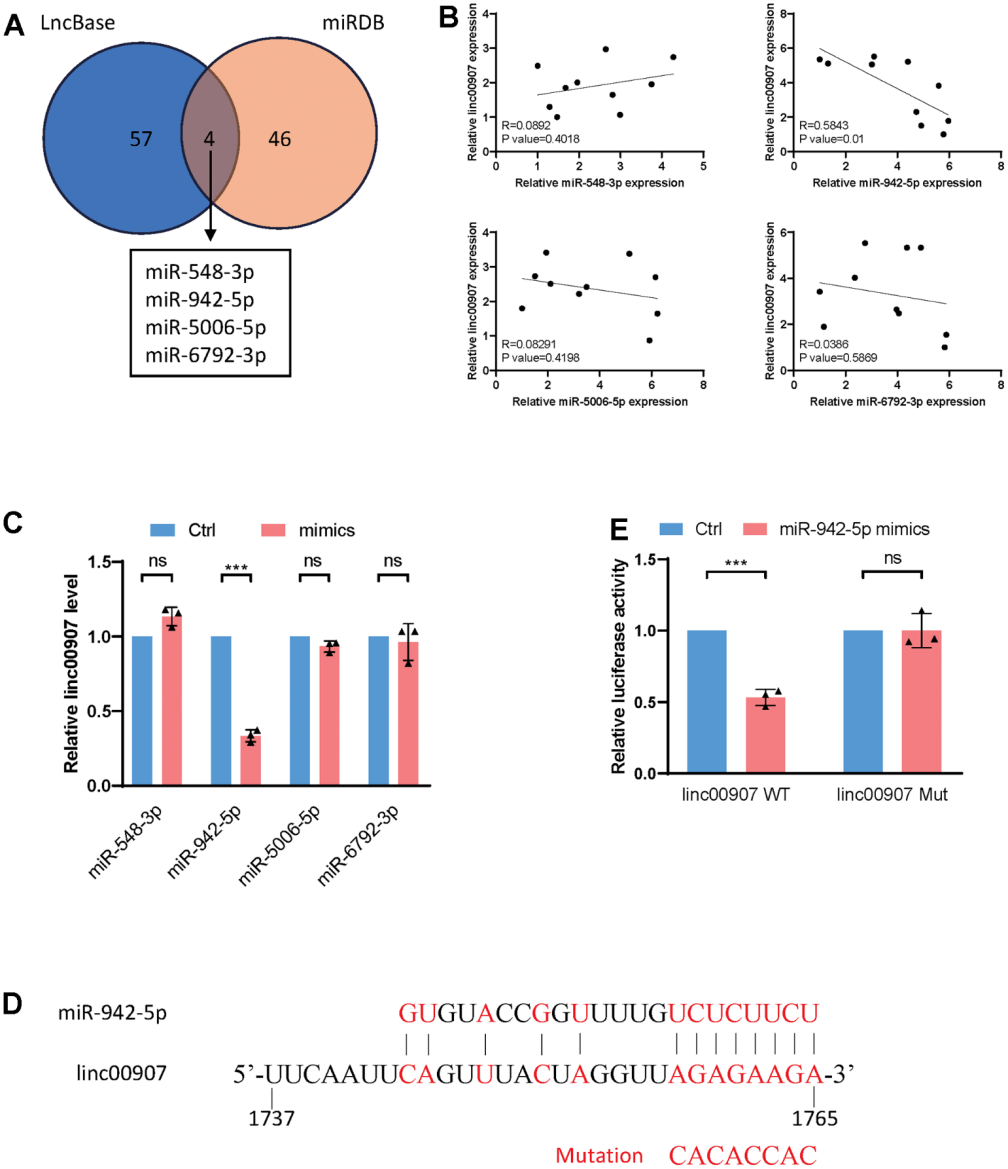
**linc00907 interacts with miR-942-5p.** (**A**) LncBase and miRDB databases were used to predict miRNAs that might bind with linc00907; (**B**) correlation analysis of expression of miRNAs with linc00907; (**C**) overexpression of miR-942-5p could downregulate linc00907; (**D**) predicted binding sequence of miR-942-5p and linc00907; (**E**) a dual-luciferase assay validated the binding of miR-942-5p and linc00907.

lincRNAs function through interactions with molecules such as proteins, miRNAs, mRNAs, or ribosomes. We hypothesized that linc00907 operates via the ceRNAs mechanism. Initially, we used the LncBase and miRDB databases to predict four miRNAs that might bind with linc00907 (Figure 5A). In ten NASH samples, we separately measured the expression levels of these miRNAs and linc00907. Correlation analysis revealed that only the expression of miR-942-5p had a negative correlation with linc00907 (R=0.5843), while the expression levels of the remaining three miRNAs showed no correlation with linc00907 (Figure 5B). Additionally, we transfected mimics of the four miRNAs and found that only the overexpression of miR-942-5p could downregulate linc00907 (Figure 5C). We predicted the specific binding sequence of miR-942-5p and linc00907 and constructed a mutant plasmid of linc00907 (Figure 5D). A dual-luciferase assay further validated that miR-942-5p could bind with and downregulate the expression level of wild-type linc00907 (linc00907 WT), but it failed to bind with the mutant linc00907 (linc00907 Mut) (Figure 5E).

### TAOK1 identified as a downstream target of miR-942-5p

**Figure 6:**
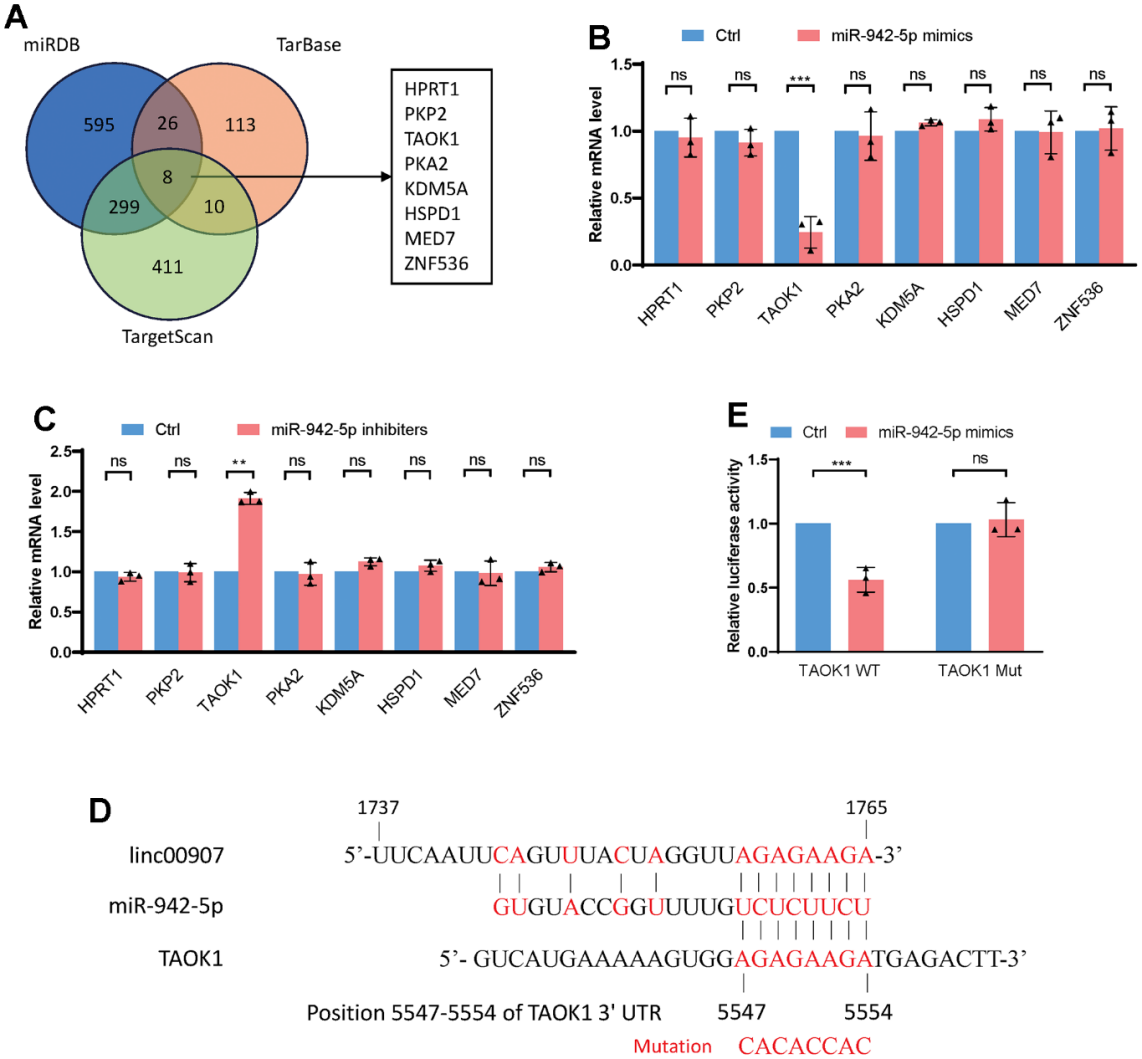
**TAOK1 identified as a downstream target of miR-942-5p.** (**A**) miRDB, TarBase, and TargetScan databases were used to predict the downstream target genes of miR-942-5p; (**B**) overexpression of miR-942-5p downregulated the mRNA levels of TAOK1; (**C**) following knockdown of miR-942-5p, an elevation in the mRNA levels of TAOK1 was observed; (**D**) the predicted specific binding sequence between miR-942-5p and TAOK1; (**E**) a dual-luciferase assay confirmed the binding of miR-942-5p and TAOK1.

We utilized three databases, namely miRDB, TarBase, and TargetScan, to predict the downstream target genes of miR-942-5p (Supplementary Tables 1–3). Upon intersecting the predictions, eight genes including HTRP1, PKP2, TAOK1, and PKA2 were shortlisted as possible downstream targets of miR-942-5p (Figure 6A). When miR-942-5p was either overexpressed or knocked down, only the mRNA level of TAOK1 changed, while the other seven genes remained stable (Figure 6B, 6C). Published studies indicate that TAOK1 is a key facilitator of inflammatory responses and lipid accumulation, which are central to the pathogenesis of NASH. Hence, we have pinpointed TAOK1 as the downstream effector of the linc00907/miR-942-5p signaling pathway [23, 24]. We predicted the specific binding sequence between miR-942-5p and TAOK1 and constructed a mutant plasmid of TAOK1 (Figure 6D). A dual-luciferase assay confirmed that miR-942-5p could bind with TAOK1-WT and downregulate its expression level, but it could not bind with the mutant version of TAOK1 (Figure 6E).

### linc00907 exacerbates NASH by elevating TAOK1 through competitive binding with miR-942-5p

**Figure 7:**
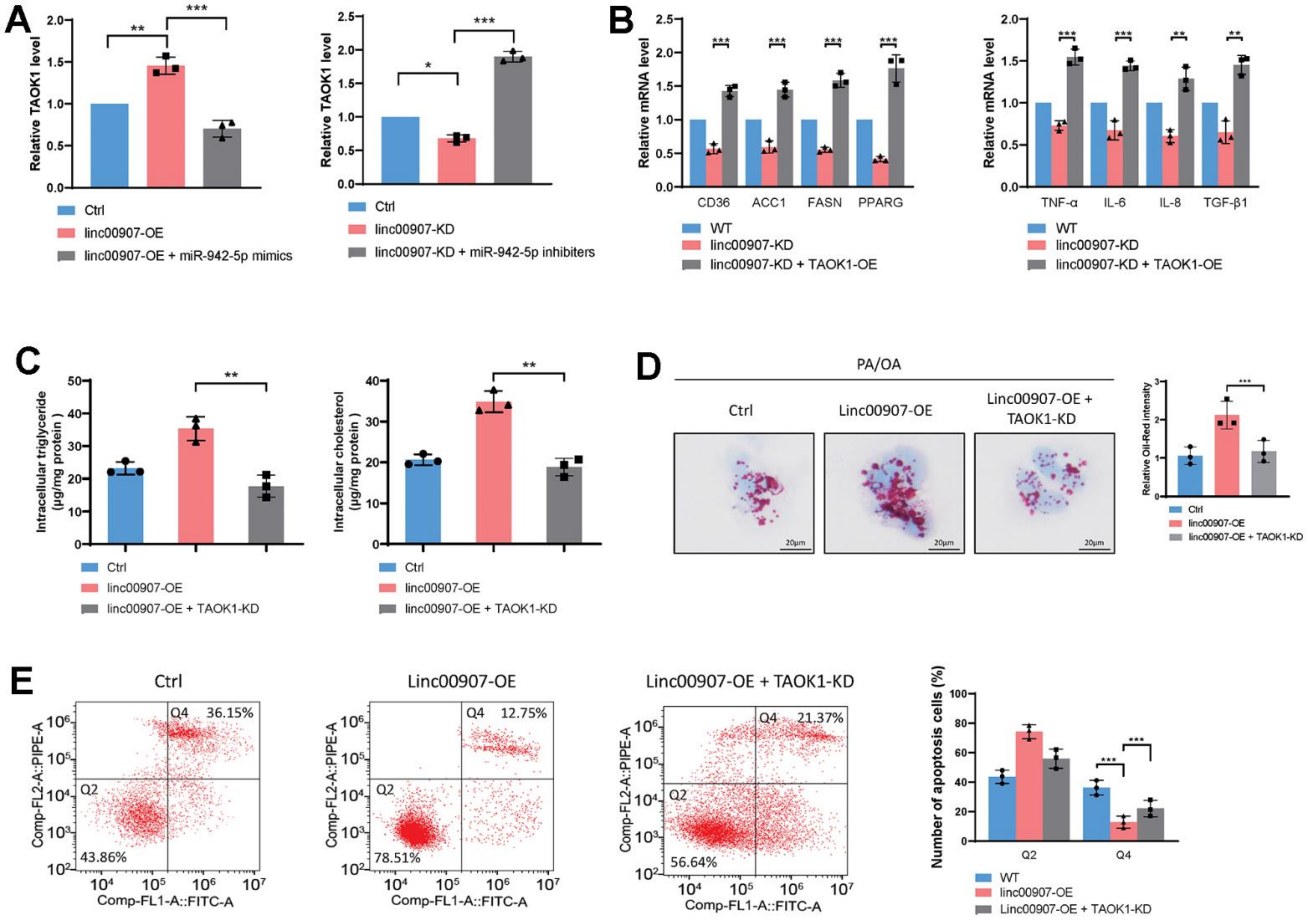
**linc00907 exacerbates NASH by elevating TAOK1 through competitive binding with miR-942-5p.** (**A**) Knockdown of linc00907 can decrease the mRNA level of TAOK1, an effect that can be countered by miR-942-5p inhibitors; (**B**) the overexpression of TAOK1 can reverse the regulatory effects on lipid metabolism-related genes and immune-related genes that result from linc00907 knockdown; (**C**) the knockdown of TAOK1 can reverse the promoting effects of linc00907 on intracellular triglyceride and cholesterol; (**D**) Oil Red O staining revealed that knockdown of TAOK1 reversed the impact of linc00907 on lipid accumulation; (**E**) linc00907 overexpression reduces apoptosis but can be reversed by TAOK1 knockdown.

Studies have indicated that TAOK1 can enhance lipid accumulation within hepatocytes, thus promoting the progression of NAFLD to NASH. We conducted rescue experiments to determine whether linc00907 promotes NASH through TAOK1. We found that overexpression of linc00907 elevates the mRNA level of TAOK1, which can be reversed by the overexpression of miR-942-5p. In contrast, knockdown of linc00907 can decrease the mRNA level of TAOK1, an effect that can be countered by miR-942-5p inhibitors (Figure 7A). Moreover, the overexpression of TAOK1 can reverse the regulatory effects on lipid metabolism-related genes and immune-related genes that result from linc00907 knockdown (Figure 7B). The knockdown of TAOK1 can reverse the promoting effects of linc00907 on intracellular lipid accumulation (Figure 7C, 7D). linc00907 overexpression reduces apoptosis but can be reversed by TAOK1 knockdown (Figure 7E). These results suggest that linc00907 competitively binds with miR-942-5p to elevate the expression of TAOK1. Furthermore, linc00907 exacerbates the progression of NASH by promoting lipid accumulation in hepatocytes through upregulation of TAOK1.

## DISCUSSION

NAFLD is a widespread liver disorder that may progress to NASH, a more severe disease linked with inflammation, hepatocellular injury, and fibrosis [25, 26]. This study aims to examine the role of lincRNA00907 in the progression of NASH. Our findings provide significant insights into the pathogenesis of NASH and potential therapeutic targets for its treatment.

Our results demonstrate a significant upregulation of lincRNA00907 in liver tissues from NASH patients, suggesting its potential association with disease progression. This finding is consistent with previous studies that have implicated dysregulation of lincRNAs in various diseases, including liver disorders.

Using *in vitro* experiments with human hepatic cell lines, we investigated the functional significance of lincRNA00907 in NASH. The overexpression of lincRNA00907 led to an increase in TAOK1 expression, which is a gene recognized to improve lipid accumulation within hepatocytes [23, 27, 28]. These findings indicate that lincRNA00907 promotes lipid accumulation in hepatocytes, potentially playing a part in the advancement and progression of NASH.

In recent years, there has been a growing interest in the functional interactions between lincRNAs and miRNAs. A negative correlation was observed between the expression levels of lincRNA00907 and miRNA-942-5p, indicating a potential ceRNA regulatory mechanism. The direct interaction between lincRNA00907 and miRNA-942-5p was confirmed using dual-luciferase reporter assays. The results suggest that lincRNA00907 can potentially serve as a ceRNA by binding to miRNA-942-5p, which in turn regulates the expression of miRNA-942-5p target genes.

Furthermore, we predicted TAOK1 as a downstream target of miRNA-942-5p and demonstrated that lincRNA00907-mediated upregulation of TAOK1 expression was reversed by miRNA-942-5p overexpression. TAOK1 is capable of inhibiting lipid catabolic processes (mitochondrial β-oxidation and triacylglycerol secretion), augmenting lipid anabolic pathways (fatty acid influx and lipogenesis), and exacerbating oxidative/endoplasmic reticulum stress, thereby intensifying lipotoxicity in hepatocytes of NASH [23]. Consequently, our research has substantiated through a comprehensive series of cellular, animal, and molecular assays that lincRNA00907 exacerbates the progression of non-alcoholic steatohepatitis (NASH) via the miR-942-5p/TAOK1 axis.

The identification of the lincRNA00907/miRNA-942-5p/TAOK1 regulatory axis provides important insights into the molecular mechanisms underlying NASH pathogenesis. Targeting this regulatory axis may offer potential therapeutic strategies for the treatment of NASH. Inhibition of lincRNA00907 or restoration of miRNA-942-5p levels could potentially reduce lipid accumulation and attenuate the progression of NASH. Additionally, targeting TAOK1 expression may represent a promising therapeutic approach to modulate lipid metabolism and inflammatory pathways implicated in NASH.

However, there are still several questions that need to be addressed. First, the mechanisms by which lincRNA00907 upregulates TAOK1 expression need to be further elucidated. It would be interesting to investigate whether lincRNA00907 directly interacts with TAOK1 protein or if it regulates TAOK1 expression through other intermediaries. Additionally, the precise functional roles of miRNA-942-5p and its downstream targets in NASH warrant further investigation. This study did not delve into the mechanism through which TAOK1 promotes the progression of NASH. Moreover, this study screened differentially expressed lincRNAs through sequencing analysis using 6 samples. The sample size is relatively small, and it can be expanded in future studies. Further studies are needed to determine the clinical relevance of the lincRNA00907/miRNA-942-5p/TAOK1 axis in NASH patients and to evaluate the potential of targeting this axis for therapeutic interventions.

In conclusion, our study provides evidence for the involvement of lincRNA00907 in the pathogenesis of NASH and its interaction with miRNA-942-5p. We demonstrate that lincRNA00907 promotes NASH progression by upregulating TAOK1 expression, leading to increased lipid accumulation in hepatocytes. Targeting the lincRNA00907/miRNA-942-5p/TAOK1 regulatory axis may hold promise for the development of novel therapeutic approaches for NASH treatment. Further investigations are warranted to validate these findings and explore the clinical implications of targeting this regulatory axis in NASH patients.

## Supplementary Material

Supplementary Table 1

Supplementary Table 2

Supplementary Table 3
